# Feeding Ecology of the Cuvier’s Gazelle (*Gazella cuvieri*, Ogilby, 1841) in the Sahara Desert

**DOI:** 10.3390/ani13040567

**Published:** 2023-02-06

**Authors:** F. Javier Herrera-Sánchez, Omar López, Javier Rodríguez-Siles, Miguel Ángel Díaz-Portero, Ángel Arredondo, Juan Manuel Sáez, Begoña Álvarez, Inmaculada Cancio, Jesús de Lucas, Joaquín Pérez, Gerardo Valenzuela, Jaime Martínez-Valderrama, Mariola Sánchez-Cerdá, Abdeljebbar Qninba, Emilio Virgós, Juan Antonio Calleja, Jordi Bartolomé, Elena Albanell, Emmanuel Serrano, Teresa Abáigar, Jose María Gil-Sánchez

**Affiliations:** 1 Harmusch, Study and Conservation of Wildlife, c/San Antón 15, 1°, Almodóvar del Campo, 13580 Ciudad Real, Spain; 2 Departamento de Zoología, Universidad de Granada, Avda. de Fuente Nueva, s/n, 18071 Granada, Spain; 3 Wildlife Ecology & Health Group (WE&H), and Servei d’ Ecopatologia de Fauna Salvatge (SEFaS), Facultat de Veterinària, Universitat Autònoma de Barcelona (UAB) Bellaterra, 08193 Barcelona, Spain; 4 Estación Experimental de Zonas Áridas, CSIC, Carretera. de Sacramento, s/n, La Cañada de San Urbano, 04120 Almería, Spain; 5 Centre de Recherche GEOPAC, Laboratoire de Géo-Biodiversité et Patrimoine Naturel, Institut Scientifique, Mohammed V University in Rabat, Av. Ibn Battota, B.P. 703, Agdal, Rabat 10090, Morocco; 6 Escet, Departamento de Biología, Geología, Física y Química Inorgánica, Universidad Rey Juan Carlos, C/Tulipán, s/n, 28933 Madrid, Spain; 7 Centro de Investigación en Biodiversidad y Cambio Global, Universidad Autónoma de Madrid, 28049 Madrid, Spain; 8 Departamento de Biología (Botánica), Universidad Autónoma de Madrid, 28049 Madrid, Spain; 9 Ecological and Forestry Applications Research Centre (CREAF), Campus de Bellaterra (UAB) Edifici C, 08193 Cerdanyola del Vallès, Spain; 10 Grup de Recerca en Remugants, Departament de Ciència Animal i dels Aliments, Universitat Autònoma de Barcelona (UAB), Travessera dels Turons, s/n, 08193 Bellaterra, Spain

**Keywords:** *Gazella cuvieri*, climatic change, diet, deserts ecology, Sahara, wild ungulates, wildlife conservation, acacia

## Abstract

**Simple Summary:**

The Sahara desert is home to the greatest diversity of ungulates of all deserts. In this harsh environment, the endangered Cuvier’s gazelle finds at the southernmost limit of its distribution a key population for its survival. A better understanding of the feeding ecology of the species may improve our understanding of the biological requirements for implementing conservation measures. In this study, we analysed the diet and feeding strategy of a desert population by combining different approaches such as faecal sampling, recording indirect signs of feeding, and direct observations of individuals. Our results revealed that Cuvier’s gazelles displayed a feeding behaviour similar to that of browsing ruminants, with acacias as key species for the survival of the species in the harsh environment of the Sahara. Consequently, the current increasing grazing pressure in remote areas and on acacias calls for measures to mitigate this emerging and possibly worsening impact due to imminent climate change.

**Abstract:**

Knowledge of the feeding ecology of ungulates in arid biomes offers an interesting model for understanding the drought resistance of large desert-adapted herbivores, a crucial issue in the face of increasing desertification due to climate change. To assess the feeding ecology of the endangered Cuvier’s gazelle (*Gazella cuvieri*) in the Sahara desert, we used a multi-method approach combining faecal samples, direct observations, and the recording of indirect signs of feeding. We hypothesised that browser behaviour is the best foraging strategy for species living in hyper-arid environments, mainly due to long periods without grazing opportunities. Complementarily, we explored the effects of the main environmental descriptors (rainfalls and NDVI) on feeding patterns and diet quality. We found that Cuvier’s diets are based mainly on acacias (*Vachellia tortilis*, *V. flava*) and occasionally on the annual forb *Anastatica hierochuntica*. In total, eighteen species (five trees, nine shrubs, three herbs, and one grass) belonging to fifteen families were recorded. Our result confirmed the browsers’ characteristic of this species, reaffirming its ability to settle in a hostile environment. Acacias stand out as key species consumed at the southernmost limit of their range; hence, future conservation plans and strategies should take this into account for the survival of Cuvier’s gazelle in desert environments.

## 1. Introduction

Deserts comprise the hyper arid regions (aridity index, AI < 0.05) of the Earth and the surrounding arid zones (AI < 0.2), occupying almost a quarter of the Earth’s surface, some 33.7 million square kilometres [1]. The scanty annual rainfall, with less of 250 mm as a rule but even complete absence in some years [2], imposes extreme conditions for the survival of any form of life. Desert-dwelling species have evolved different physiological and behavioural strategies in the face of scarce water availability and extreme exposure to solar radiation. These strategies may even differ according to the size of the animal, as in the case of burrowing desert rodents or the heterothermy of arid-adapted ungulates, which allows them to survive in harsh desert environments. In this context, adaptations in terms of food quality and active foraging in relation to their trophic ecology may further condition the type of survival strategies in deserts, including for obtaining water under extreme conditions [3]. Unfortunately, large desert mammals not only have to cope with the harsh desert conditions, but they are also mostly endangered in the Sahara with extreme cases such as the critically endangered addax (*Addax nasomaculatus* [4]) or the critically endangered Saharan cheetah (*Acinonyx jubatus hecki* [5]). The Sahara desert is home to the highest diversity of antelope species of any desert ecoregions in the world, namely the scimitar-horned oryx (*Oryx dammah*), addax, dama gazelles (*Nanger dama*), slender-horned gazelles (*Gazella leptoceros*), Cuvier’s gazelles (*G. cuvieri*), and Dorcas gazelles (*G. dorcas*) [6,7]. The recent decline in these populations has been due to overhunting, and habitat destruction [6] has not been followed by an increase in the research efforts on existing populations to develop conservation plans [8,9,10], most likely due to the hostile conditions of their habitats and the political instability that makes conditions unsafe for researchers [11]. However, knowledge of the ecology of wild ungulates in arid environments is a hot topic in the current climatic change scenario [12]. In this sense, understanding the feeding strategies of large mammals in desert biomes becomes a key goal.

Desert environments are characterised by low and variable rainfall (both spatially and temporally), resulting in minimal and unpredictable pulses of primary production lasting for a few weeks [3,13]. In this environment, herbivores must adapt their foraging strategy to the high uncertainty of food availability and its quality. The quality provided by the plants is deduced from the concentration of nitrogen and fibre which are measured by different methods. Two common procedures are direct observation of consumption in certain plants for which the chemical composition is known and detailed analysis of faecal composition. Both tend to show that woody plants are a poorer-quality source compared to fresh herbaceous taxa [3,13,14]. Similarly, the quality of the diet can be largely determined by the environmental conditions that, in arid and desert environments, greatly limit the abundance of vegetation and productivity [3,13]. In fact, deserts usually host scattered sclerophyllous or deciduous woody plants, often spiny or/and with tiny leaves, low palatable perennial graminoids, and annual plants with a phenology dependent on erratic rainfall [3,13]. 

In relation to the preference for feeding on herbaceous plants or the ability to consume woody plants, ruminants are conventionally classified as “grazers” with a mainly bulk/grass diet, “browsers” with a concentrated diet of browsers or herbs, and “intermediate” for opportunists and mixed feeders [15,16]. In addition, some physiological adaptations make ungulates more energetically efficient at grazing or browsing [14]. In semi-arid and desert environments, browsing species become an important component of the diet during hot and dry summers, as a rapid decline appears in quality and herbaceous availability [2]. The alternation between grazing and browsing is well known and depends on the frequency and intensity of rainfall as well as the extent of grazing for many species (e.g., Eland (*Taurotragus oryx*) [17], Dorcas gazelle [18], Dama gazelle (*Nanger dama*) [8], and Mountain gazelle (*Gazella gazella*) [19]). Hence, to survive in deserts, ruminants could alternate between the following feeding behaviours: (1) grazing opportunistically to take advantage of eventual pulses of ephemeral grasses and forbs that grow after occasional rainfalls, possibly in conjunction with nomadic movements (e.g., the case of addax [20]); or (2) browsing on more stable resources such as perennial shrubs and trees growing in desert massifs. This second strategy could be the selected one for long-term survival in the case of sedentary species that have to endure long periods of drought that preclude grazing. This scenario might be the case of the Cuvier’s gazelle, as its Saharan population is strictly restricted to the main massifs [10,21] with no opportunities for long migrations.

Cuvier’s gazelle is an ideal model for studying the survival strategies of ruminants in deserts. This medium-sized ungulate is endemic to northwestern Africa [22]. However, over the last century, long-term overhunting and recent habitat loss have drastically reduced its population sizes and subsequent assessments of extinction risk as a vulnerable species [23]. The species occupies a wide range of habitats from Mediterranean scrubland and open Aleppo pines (*Pinus halepensis*), arar tree (*Tetraclinis articulata*), oaks (*Quercus* spp.) and junipers (*Juniperus* spp.) to the Sahara desert [22,24]. However, the extant populations are now restricted to small and isolated patches in mountainous terrain in the Maghreb highlands of Morocco, Algeria, and Tunisia, and also in arid Mediterranean steppes and the Atlantic Sahara desert [22,25]. The Atlantic Sahara desert is one of the last strongholds of the endangered Cuvier’s gazelle [10,21]. Here, it reaches its southernmost distribution, inhabiting biotopes very different from the typical landscapes it occupies in the Atlas Mountains, where the climatic conditions give rise to more productive environments than in the Sahara [22]. Therefore, a better understanding of the adaptive traits that enable the survival of this species in this hyper-arid environment is crucial to implement effective conservation measures [10,25,26]. However, the current scientific knowledge on the diet of Cuvier’s gazelle is very scarce. Only two studies on free-ranging gazelles have been published but restricted to the Mediterranean population of Djebel Messaâd Forest, Algeria [27,28], and another one has focused on a reintroduced group in a small fenced area in Tergou reserve, Morocco [29]. 

In this study, we analyse the feeding strategy of Cuvier’s gazelle in the Sahara. In particular, we examine the diet of Cuvier’s gazelle and its components that could play a key role in their survival and, thus, their ability to be sedentary in a Saharan environment. We hypothesise that the foraging strategy of Cuvier’s gazelle in this environment is mainly based on browsing, due to both the long periods without rain and its proximity to mountains where trees and bushes are common [10]. To achieve our objective, we use a multi-method approach combining faecal-based analysis, direct observations of foraging behaviour, and recording indirect signs of feeding. Secondly, we explore the relationships between key environmental descriptors (precipitation, primary production assessed through the NDVI, altitude, and temperature), the observed consumption patterns, and diet quality. Finally, beyond improving the knowledge of our targeted species, we provide baselines that could be useful for studying the feeding ecology of other wild ungulates in remote and harsh deserts environments.

## 2. Materials and Methods

### 2.1. Study Area

**Figure 1:**
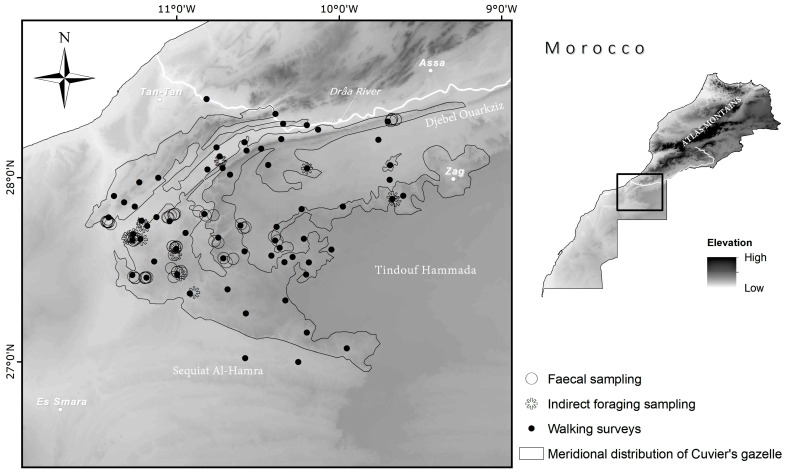
Study area with sampling sites. Relief is represented by a raster digital elevation model (DEM) and is expressed by a colour gradient from darker (higher altitude) to lighter (lower altitude). The map also shows the main towns and distribution of Cuvier’s gazelle south of the Drâa River, considered its southernmost range [10,21].

We studied the diet of Cuvier’s gazelles in a vast region between the lower Draa River and the upper basin of the Sequiat Al Hamra, within the Atlantic Sahara of Morocco (Figure 1). The study area extends over 20,000 km^2^ and is a typical Saharan landscape, with a subtropical desert at low-latitude (Köppen–Geiger classification [30]) and hyper-arid climate [1]. The area falls inside the North Saharan Xeric steppe and woodland ecoregion with prolonged droughts and irregular rainfalls [31]. Mean, minimum, and maximum temperatures are 22.7, 8.0, and 39.0 °C in the western zones (closer to the Atlantic Ocean); 23.2, 0.0, and 43.0 °C in the eastern zones; 19.1, 10.7, and 29.0 °C at the northern limit, respectively. Total annual precipitation (with large annual variability) is 138, 59, and 190 mm, respectively (recorded at climate stations at Smara, 26°46′ N, 11°31′ W; Tindouf, 27°40′ N, 8°7′ W; Tan Tan, 28°26′ N, 11° 06′ W).

The study area is typically mountainous with rocky plateaus and a complex network dry rivers of which only the Draa and Chebeiqa rivers can maintain permanents pools of water. The whole region is a transition point between the Saharan and Macaronesian ecoregions [2,32,33]. In this context, the presence of plant species such as the argan tree (*Argania spinosa*), a Moroccan endemism, and the presence of Macaronesian elements such as *Euphorbia officinarum* are noteworthy but very limited to some mountain refuges. Vegetation is very sparse and is mainly associated with mountainous areas and ravines that favour higher soil humidity. Acacias (*Vachellia tortilis* subsp. *raddiana* and *V. flava*) along with some scattered specimens of *Balanites aegyptiaca*, *Maerua crassifolia*, and *Calotropis procera* form the dominant tree vegetation. *Periploca laevigata*, *Launaea arborescens*, *Searsia tripartita*, *Nitraria retusa*, *Saharanthus ifniensis*, *Salsola tetragona,* and *Lycium shawii* stand out as shrub vegetation, and *Cymbopogon schoenanthus* as grass. Other species that thrive mainly on hammadas (*plateaux*), regs (*stony plains*), and ergs (*dune areas*) are *Anabasis articulata*, *Hammada scoparia*, *Helianthemum lippii*, and *Nucularia perrinii* among perennial shrubs, and *Anastatica hierochuntica*, *Citrullus colocynthis*, *Mesembryanthemum cryptanthum*, *Cullen plicatum*, *Panicum turgidum*, *Stipagrostis plumosa*, and *Pennisetum divisum* among ephemeral forbs and perennial grasses that experience short-term pulses, lasting for a few months (or weeks) only during the years with enough rainfall [34].

As a result of a massive general decline in the region’s large vertebrate fauna [6] and following the last sightings of the Dama gazelle after the mid-20th century, there are currently three ungulates remaining in the study area: Dorcas gazelle, Barbary sheep (*Ammotragus lervia*), and Cuvier’s gazelle [35]. Here, Cuvier’s gazelle population is one of the most important, if not the most, of its global range, with an estimated 1000 individuals distributed over 12,000 km^2^, resulting in a very low density of 0.08 ± 0.02 individuals per square kilometre [21]. This population is severely affected by poaching and habitat destruction related to overgrazing by goat and sheep, habitat fragmentation due to roads and water cisterns construction, and timber harvesting [36].

### 2.2. Data Collection

First, it is important to take into account two main limitations of our survey in the study area: (1) the remoteness, which includes high limitations for displacements due to the scarcity of roads, mostly represented by unpaved roads only accessible by 4WD vehicles; (2) the very low density of Cuvier’s gazelle. To overcome both challenges, we accomplished walking surveys to collect samples for three types of data: faecal samples, direct observations, and indirect records. Fieldwork was carried out during 18 expeditions with a maximum of 15 persons per expedition, conducting 67 sampling points every March–April and December–January annually from 2012 to 2019, to avoid the harsh conditions during the rest of the year (temperatures > 45 °C); the other four expeditions were during May (2), September (1), and October (1). Each study site was located in the different habitats of the study area to make a spatially independent distribution covering the entire area of interest. In a typical walking survey (12.08 ± 0.72 km by 2–3 persons), we looked for sightings of individuals and indirect signs such as footprints, scats, and signs of grazing/browsing activity (see further details in [21]). The long sampling period allowed us to capture the typical rainfall variability of the study area.

**Figure 2:**
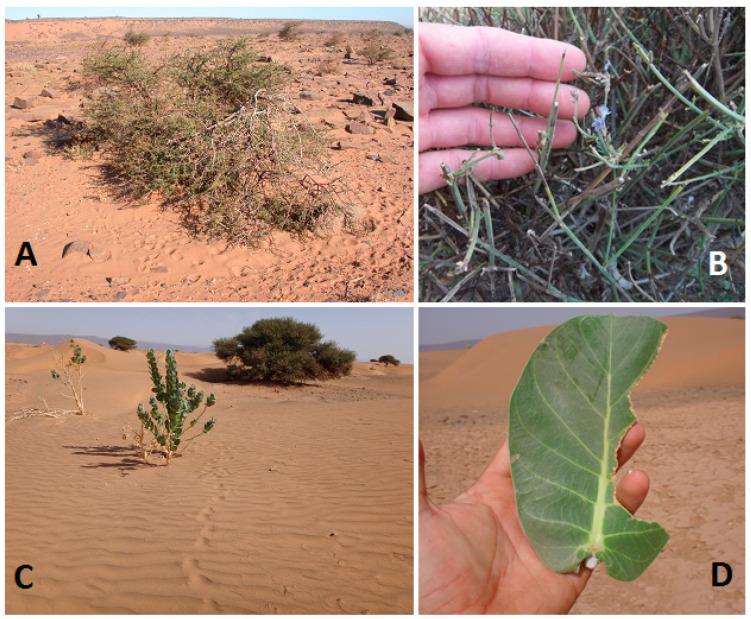
Examples of Cuvier’s gazelle indirect feeding (IF) data obtained in the study area during the surveys: (**A**) Footprints recorded while one group was feeding on *Vachellia tortilis*. (**B**) Damage in *Lavandula coronopifolia* by browsing. (**C**) *Calotropis procera* consumed by a Cuvier’s gazelle. (**D**) Leaf of *C. procera* partially consumed by Cuvier’s gazelle.

Faecal samples (FSs) provide a good approximation of the diet of ungulates [37], including desert gazelles [27,38]. The collected FSs were confirmed as belonging to Cuvier’s gazelle by genetic analysis (see details in [39]). During field surveys, foraging behaviour was recorded by direct observation (DO) of individuals using binoculars or spotting scopes of high-magnification. Plants that were eaten and those that were visited but not consumed were counted (see [40,41] for another desert gazelle species). Indirect signs of feeding (IF) were recorded from the detected fresh tracks of Cuvier’s gazelle using footprints in sandy terrain, which were followed for as long as possible. Plants with associated tracks (see examples in Figure 2A,B) were examined in situ for fresh bites on leaves and branches recording the species consumed (Figure 2) (see [42] for IF applied on Addax or [43,44] on moose (*Alces alces*) by tracking in the snow). Plants < 2 m from the track were considered ignored and, therefore, not consumed. The tracks of the smaller Dorcas gazelle may be confused with those of our target species, but this gazelle is almost absent in our study area in areas inhabited by Cuvier’s gazelle (data from our surveys); in any case, tracks < 5 cm in length of isolated individuals were rejected [45]. As for domestic ungulates, both the number of tracks left and the clear morphological differences of the footprints (as for Barbary sheep) were easily identified and discarded.

### 2.3. Faecal Cuticle Microhistological Analysis of FSs

For the analysis of FSs, we used microhistological techniques based on the analysis of the plant cuticles, following [46]. This technique is widely used to determine the diet of herbivorous mammals, e.g., [46,47,48], including our target species [27,28]. We added 1 g of faecal samples in 5 mL of HNO_3_, concentrated in test tubes, and this preparation was introduced in a hot water bath (80 °C) for 1 min. After this time, the content of these tubes was poured into 200 mL of distilled water. This solution was passed through a 0.125 mm filter to separate the liquid from the solid sample. To prepare the slides, we added 3 drops of 50% aqueous glycerine solution; then, we took the solid that had remained on the filter and added it to the slide, distributing everything homogeneously. When everything was homogenised, we mounted the coverslip with DPX microhistological varnish. All these samples were prepared in duplicate. We let the samples dry for one or two days and then performed the analysis with a microscope. The samples were examined under 10–40× magnification. One hundred plant fragments were counted per slide and identified to species level on the basis of epidermis and trichomes morphology [49]. 

### 2.4. Diet Quality 

Diet quality was assessed through faecal nitrogen (FN), which is widely used in diet quality assessment [14,50,51]. In addition, the use of neutral detergent fibre (NDF = Cellulose + Hemicellulose + Lignin), acid detergent fibre (ADF = Cellulose + Lignin), and acid detergent lignin (ADL = lignin) is useful to complement the information given by FN as both follow opposite patterns [52,53]. As FN is the combination of metabolic and food residue nitrogen excreted in faeces, FN/NDF, hereafter called FNc, was calculated as a proxy for the protein contents of diets [52]. Near-Infrared Spectrophotometry (NIRS) was used to predict faecal nitrogen and fibre contents [51]. Briefly, the samples were placed in 35 mm diameter quartz beakers and analysed in a NIRSystem 5000 spectrometer (FOSS, Hillerød, Denmark) using wavelengths from 1100 to 2500 nm. Data were obtained at 2 nm intervals as log 1/R, where R is the reflectance. Each sample was scanned in duplicate by making a 180° turn of the glass. If the amount of sample was too small, the base of the glass was not completely covered, but a beaker was added to reduce the surface by half so that two readings could be taken, each with its duplicate. Faecal nitrogen data were obtained using the multispecies equation [51].

Subsequently, faecal nitrogen was corrected with neutral detergent fibre [52]. For fibre content, 10 samples were analysed in the laboratory following the Van Soest method [54] using the Ankom 200 Fiber Analyzer (ANKOM Technology, Macedon, NY, USA). Afterwards, the values obtained from the 10 samples were used to readjust the NIRS equation and to obtain more accurate data due to the correction provided by this method. 

### 2.5. Environmental Descriptors 

We selected six environmental descriptors that could affect the foraging behaviour of Cuvier’s gazelles: normalised difference vegetation index NDVI (per year, seasonal, and weekly scales), annual precipitation, annual temperature, and altitude. The values of the NDVI index were extracted from the map of the USGS Land Cover Institute (LCI) website (available at https://earthexplorer.usgs.gov/; accessed on 15 September 2022). Climatic variables and elevation data were taken from the WorldClim–Global Climate Data (available at http://worldclim.com/version2; accessed on 14 June 2022) and the DIVA-GIS websites (available at https://www.diva-gis.org; accessed on 3 May 2022), respectively.

### 2.6. Data Analyses

As we were only able to observe very few individuals feeding, this type of data was not used for statistical analysis. For the other two approaches, we first compared the information obtained by FSs and IF through (1) the taxonomic richness (number of consumed species) and (2) the contribution (% of occurrence) of each consumed plant, using Chi^2^ tests on 2 × 2 contingency tables with Bonferroni’s correction to detect significant differences. Each consumed species was classified as woody species (trees and shrubs), forbs, or grasses (Table 1), to define the feeding strategy resulting from each methodological approach (i.e., browser, grasser, or intermediate).

Second, plant species selection was examined using the IF data set; for this, we compared the contribution of consumed species with the contribution of non-consumed species detected for each monitored trackway. We pooled all data on a single sample and calculated the Ivlev’s selectivity index [55], also tested by Chi^2^ tests on 2 × 2 tables with Bonferroni’s correction.

Third, we used the FS data to explore the multiple relationships between diet composition (proportion of consumed plants), diet quality (FNc, NDF, ADF, and ADL), and environmental descriptors (rainfalls and NDVI). We began by exploring the relationships between diet quality descriptors, the occurrence of consumed species, and some environmental variables through Pearson’s correlations. Then, we carried out a Principal Component Analysis (PCA) [56]. In our PCA modelling, the dietary components of gazelles (plants and the faecal indicators of diet quality) were used to build the PCA dimensions (active variables), whereas the descriptors for the environmental variation were considered as supplementary quantitative variables. The Shannon’s diversity index was also included in the set of active variables. PCA was performed in the FactoMineR 1.63 version [57] package of the statistical software R 4.2.1 version (R Core Team 2022).

## 3. Results

### 3.1. Diet Composition and Feeding Behaviour

**Figure 3:**
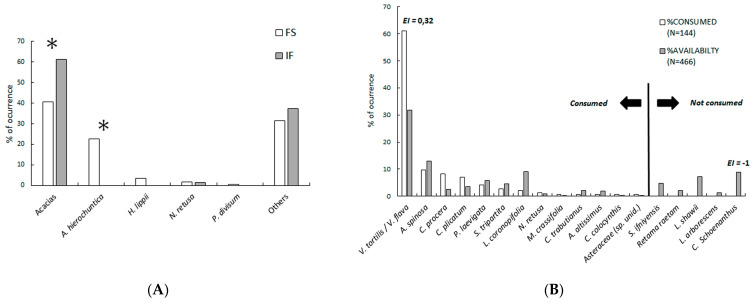
(**A**) Contribution of plant species to the diet of Cuvier’s gazelles in the Sahara desert: from faecal samples (FSs) and indirect feeding (IF) data; * *p* < 0.001 of Chi^2^ tests with Bonferroni’s correction. (**B**) Diet selection from IF; Ivlev’s electivity index (*EI*) is shown only for species with *p* < 0.0027 of Chi^2^ tests with Bonferroni’s correction for acacias (*Vachellia tortilis* and *V. flava*) and *Cymbopogon schoenanthus*.

The IF after 15 tracks (mean length 624 m, range 300–1600 m) provided information on at least 27 gazelle individuals (11 tracks during April, and 1 track during December, January, March, and September). These IF records were made on 144 individual plant species and 14 species of trees and shrubs (Table 1, Figure 1 and Figure 3). On four independent occasions, seven gazelles were directly observed feeding on *P. laevigata* (1), *L. shawii* (1), *Asparagus altissimus* (8), *V. flava* (6), and other unidentified species (twelve times, probably small forbs), amounting to at least 4 species and 28 individuals. We obtained 57 samples (independent groups of pellets) for FS analysis (Figure 1), 21 collected during December–January and 36 during March–April. The FS analysis provided 5766 cell fragments with acacias being the best represented with a total of 2337 fragments (40.54%), followed by unidentified plant taxa with 1808 fragments (31.37%), *A. hierochuntica* with 1298 fragments (22.51%), *H. lippii* with 206 fragments (3.57%), *N. retusa* with of 93 fragments (1.61%), and *P. divisum* with 23 fragments (0.40%). Therefore, only five or six species were identified from the faecal samples (the two acacia species could not be differentiated). It is remarkable that *A. hierochuntica* was not detected by tracking despite its contribution to the diet (Figure 3). In our study area, Cuvier’s gazelles feed on eighteen species (five trees, nine shrubs, three forbs, and one grass), belonging to fifteen families (Table 1, Figure 3). The percentage of trees and shrubs depends on the method of analysis used and varies between 45.72% for FS, 57.1% for DO, and 98.6% for IF (Figure 3). 

There were important differences in diet composition between the FS and the IF data sets. The IF offered a higher contribution of trees, a null contribution of *A. hierochuntica*, and a lower contribution of the other species (Figure 3A). In terms of taxonomic selection, the tracked Cuvier’s gazelles showed a preference for acacias while they tended to consume all other species according to their availability, except for the grass *Cymbopogon schoenanthus*, which was neglected by the monitored gazelles (Figure 3B). A group of three gazelles tracked over 1.6 km did not consume ripe argan fruits in fifteen available trees.

### 3.2. Effects of Environmental Factors 

**Figure 4:**
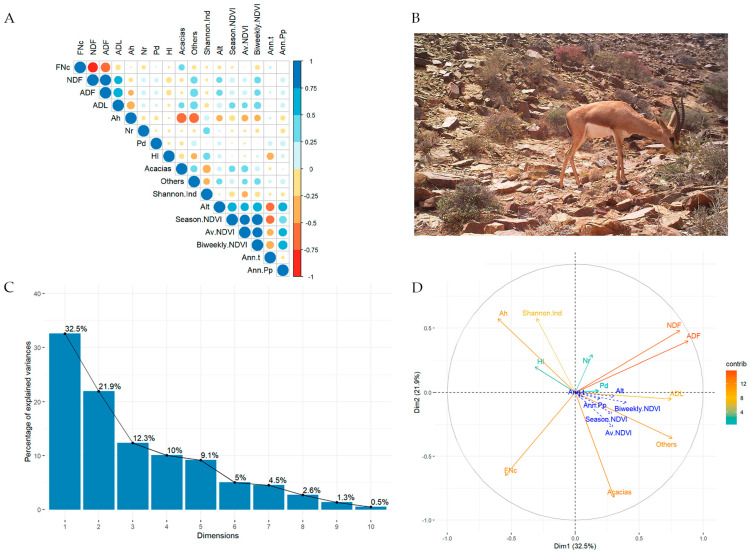
(**A**) Correlation panel showing Pearson’s coefficients between pairs of variables; positive relationships are represented in blue, negative ones in red. (**B**) Photo capture by camera-trapping of a male Cuvier’s gazelle feeding on *Asparagus altissimus* in the study area. (**C**) Bar plot representing the proportion of variance explained by the dimensions extracted from the PCA. (**D**) PCA results for relationships between the species consumed, Shannon’s index, diet quality, and selected environmental factors. Abbreviations: plants consumed: Ah (*Anastatica hierochuntica*), Nr (*Nitraria retusa*), Pd (*Pennisetum divisum*), Hl (*Helianthemum lippii*); Alt (Altitude), Annual temperature (Ann.t), Annual precipitation (Ann.Pp); NDVI: per year (Av.NDVI), weekly scales (Biweekly.NDVI), and seasonal (Season.NDVI); Shannon’s diversity index (Sannon.Ind). For all other abbreviations, see text.

A summary of diet composition and diet quality from FSs is shown in Table 2. Eleven PCA components were obtained, but the first two explained more than half (54.43%) of the variability observed in the dietary elements (Figure 4C). In the first PCA component (which retains 32.5% of the observed variability), the variables with the highest contribution were ADF and NDF fibre, followed by the proportion of unidentified plants and ADL fibre. In contrast, the lowest contribution was for the proportions of *P. divisum* and *N. retusa*. The second component accounted for 21.9% of the observed variability in our set of diet variables and was mainly represented by the proportion of acacias, the diet quality, and the Shannon index. The proportion of *A. hierochuntica* and NDF and ADF fibres were also important for the second PCA component. The PCA correlation circle (Figure 4D, see data in Supplementary Material Table S1) reflects a relatively complex net of relationships with the following remarkable results: (1) for diet quality descriptors, fibres showed a negative relationship with FNc (as expected), acacias consumption was weakly associated with FNc, and fibres were associated with the “Others” category and negatively associated with *A. hierochuntica*. (2) As for environmental descriptors, primary production at different time intervals (annual, seasonal, and weekly), annual precipitation, annual temperature, and altitude had very little relationship with plant consumption or with faecal indicators of diet quality. (3) In terms of relationships between the consumed species, *A. hierochuntica* was negatively correlated with acacias and with the “Others” category, and trophic diversity was negatively related to acacias. 

## 4. Discussion

### 4.1. Limitations of the Study

Our results highlight the great difficulties of wildlife research in the Sahara. First, we obtained a relatively low sample size for IF and DO after a great effort surely due to the very low density of Cuvier’s gazelles [21], their shy behaviour resulting from constant poaching (confirmed during field surveys), and the limitations for fieldwork in such a rocky terrain. The latter circumstance determined the non-detection by IF of important species for the feeding ecology in the Sahara, as was the case of *A. hierochuntica*, a widespread annual forb in the study area. However, IF resulted in a significantly higher number of identified consumed plants and, in addition, this method allowed the only approach to food selection in a free-ranging population of Cuvier’s gazelles of which we are aware, confirming a strong preference for acacia species as a key result. 

In contrast to DO and FI, FSs were relatively easy to find [21], but we obtained a very low identification success of species consumed, which was a handicap for the description of the foraging behaviour (i.e., for testing our hypothesis). This result contrasts with the FS analyses by Benamor et al. [27,28], who obtained a higher identification success in FSs of Cuvier’s gazelles in a Mediterranean population. The discrepancy may be partly explained by: (1) the large differences detected in the taxonomic composition of the diet between the Saharan and the Mediterranean studies [27], and/or (2) differences in the degree of field preservation of collected pellets. Although this problem may be solved through DNA identification of the plants consumed (see, e.g., the case of Dorcas gazelle [38]), another limitation was the expensive costs of genetic analyses; in fact, we obtained many more samples (see [21]) that could not be used, due to lack of budget for laboratory work.

Poor success in identifying the species consumed by FSs limited the correct classification of the foraging strategy, and DOs were inconclusive due to the low sample size. The IF method was much more capable of classifying the foraging behaviour of Cuvier’s gazelle as a browser than of revealing its diet composition. 

Finally, our samples were biased to December–January and March–April, particularly due to the limitations of surveying the rest of the year in such a hostile environment. In any case, this circumstance did not affect the prediction for our main hypothesis (that is, Cuvier’s gazelles are browsers in the Sahara) as we sampled during the seasons of maximum rainfall and, thus, during the maximum availability of herbs and grasses. However, this seasonal sampling bias could affect the relationships between diet and environmental descriptors, so these results should be assumed to be representative of winter and spring only.

### 4.2. Feeding Strategy

Following our hypothesis, the results suggest that Cuvier`s gazelles are mainly browsers in the Sahara desert, showing a strong preference for trees, as can be deduced from (1) the resource selection patterns observed in the analysis of IF data, and (2) the negative relationship between this plant category and diet diversity in the analysis of FS data, as predicted by the optimal foraging theory [58,59,60,61]. Moreover, the tracked gazelles avoided some available grass species, even when these plants were growing after rainfall (the case of *C. schoenanthus*). Cuvier`s gazelles had previously been considered as an intermediate feeder (i.e., browser-grazer) based on a study carried out in their Mediterranean range, where the diet is totally different from that of the Sahara, with *Stipa tenacissima* grass and the *Artemisia herba-alba* shrub as the predominant food items among the 29 species of consumed plants [27,28]. Therefore, this ungulate is an adaptable ruminant that switches its foraging behaviour according to plant availability, following a typical opportunistic feeding strategy [60,61]. In the Atlantic Sahara, the other *Gazella* species present is the smaller Dorcas gazelle, which seems to be a browser-grazer intermediate in this region, basing its diet on acacias and the succulent species *N. perrinii* and *M. cryptanthum* [62]. This strategy is probably favoured by nomadic movements following rain pulses [63], allowing Dorcas gazelles to have a much wider distribution than Cuvier’s gazelles in the Atlantic Sahara, as they also inhabited the vast flat regions south of the study area, where our targeted species is absent [35]. 

The presence of trees such as acacias and argan trees plays a key role in the survival of Cuvier`s gazelles in the case of the Sahara desert and, in particular, acacias as the most consumed and selected tree species. In fact, this genus also fulfils an essential role in the diets of Sahelo-Saharan antelopes [20] and other desert gazelles such as Acacia gazelle (*G. acaciae*) and Arabian Desert gazelle (*G. cora*), especially *V. tortilis* [64,65]. Acacias belong to the family Leguminosae, a group with a high nitrogen content and, therefore, probably with higher nutritional quality than other Saharan species. IF showed that acacias were more selected compared to argan, although the latter tree species also plays an important role in the local feeding ecology of Cuvier’s gazelles. However, our IF data were inconclusive due to the small sample size, and did not confirm the consumption of the fruit of this tree by Cuvier`s gazelles, as has been previously mentioned [35].

*A. hierochuntica* was the most important alternative species after trees, supporting the opportunistic condition of Cuvier`s gazelles. The contribution of this annual forb to the diet suggests a key role during drought periods, an aspect discussed below. The consumption of *C. procera*, previously cited for Arabian Desert gazelles [65], is an interesting result as this tree is a highly toxic species, particularly its latex [66]. Gazelles only fed on one or two leaves per visited tree, taking only a few pieces of each leaf (see Figure 2). This behaviour may allow the avoidance of latex secreted by the plant in response to browsing bites. In this respect, there is some risk to the gazelles, but it is probably worth the reward, as *C. procera* has one of the broadest leaves of all Saharan plant species, rich in water content.

We found only weak effects of primary production on observed foraging patterns and diet quality during winter and spring. The rugged habitat of the study area provides sheltering conditions for trees and shrubs at the micro-habitat level by maintaining humidity in ravines and canyons, and reducing evapotranspiration due to the shade provided by the relief, thus helping to mitigate periods of drought [3,67]. This scenario favours the resilience of Cuvier´s gazelles to Saharan conditions, thanks to a relatively constant food supply, which would partly explain the habitat selection patterns observed in this region [10]. The results only allow us to hypothesise that gazelles would probably increase forbs consumption during periods of drought (lower NDVI), as an adaptive response to low food availability. During periods of drought, Saharan woody species adopt a deciduous strategy by removing most, if not all, leaves in extreme situations [68]. This scenario probably forces gazelles to increase their foraging effort, consuming species that should be avoided when primary production is higher, such as *A. hierochuntica* and the toxic *C. procera*.

## 5. Conclusions

There are important limitations to the correct description of the Cuvier’s gazelle diet in desert environments if only FS approaches are used. Therefore, further research is needed to determine the reasons for this low success of plant tissues identification. In the meantime, in the absence of optimal resources to carry out genetic approaches, we recommend combining all available non-invasive techniques to maximise the dataset in the study of remote areas, especially considering the logistical constrains that make it challenging to study wild ungulates in such regions. 

Moreover, we detected strong evidence of foraging strategies that allow Cuvier’s gazelles to live at the edge of their optimal habitat conditions. Through our combined approaches, we confirmed the marked plasticity of this species, which adapted to browser behaviour in the stressful conditions of the Sahara desert. In this sense, the presence of acacias probably represents the last resort for its survival there, being a key result for the design of correct conservation strategies for this threatened ungulate. An immediate recommendation applied to the conservation of this key population is to urgently study the impact on the trees of the large number of herds of goat and sheep that invade this region during the rainy season. This emergent problem is related to the improvement of trucks transport thanks to new roads and the construction of a considerable network of water cisterns, resulting in intensive land use that is very different from the traditional nomadic pastoralism of the Atlantic Sahara. Lastly, the observed patterns of feeding ecology of Cuvier’s gazelles in desert conditions provide an interesting model for understanding the drought resistance of desert-adapted large herbivore species, an issue that will be crucial in the face of increasing desertification due to climate change.

## Figures and Tables

**Table 1:** Species consumed by Cuvier’s gazelles, category and detection method.

Species	Family	Category	Method ^1^
*Vachellia flava*	Leguminosae	Tree	DO, IF, FS
*V. tortilis* subsp. *raddiana*	Leguminosae	Tree	IF, FS
*Anastatica hierochuntica*	Brassicaceae	Forbs	FS
*Argania spinosa*	Sapotaceae	Tree	IF
*Asparagus altissimus*	Asparagaceae	Shrub	DO, IF
Asteraceae (sp. unidentified)	Asteraceae	Forbs	IF
*Calotropis procera*	Apocynaceae	Tree	IF
*Citrullus colocynthis*	Cucurbitaceae	Forbs	IF
*Convolvulus trabutianus*	Convolvulaceae	Shrub	IF
*Cullen plicatum*	Leguminosae	Shrub	IF
*Helianthemum lippii*	Cistaceae	Shrub	FS
*Lavandula coronopifolia*	Lamiaceae	Shrub	IF
*Lycium shawii*	Solanaceae	Shrub	DO
*Maerua crassifolia*	Capparaceae	Tree	IF
*Nitraria retusa*	Nitrariaceae	Shrub	IF, FS
*Pennisetum divisum*	Poaceae	Grass	FS
*Periploca laevigata*	Apocynaceae	Shrub	DO, IF
*Searsia tripartita*	Anacardiaceae	Shrub	IF

^1^ Direct observations (DOs), indirect data of feeding recorded from fresh tracks (IF), and faecal samples of Cuvier’s gazelles (FSs).

**Table 2:** Mean ± SD, minimum, and maximum concentrations (%) of the assessed items in 57 faecal samples from Cuvier’s gazelles collected in December, January, March, and April from 2012 to 2019 in the Sahara desert, Morocco.

	Faecal Items ^1^	Mean ± SD	Range
**Diet composition**	*Anastatica hierochuntica*	22.77 ± 23.80	0–80.00
	Acacias	41.01 ± 16.42	9.00–82.00
	Others	31.73 ± 13.9	6.00–71.00
	*Helianthemum lippii*	3.61 ± 8.18	0–40.00
	*Nitraria retusa*	1.63 ± 4.72	0–33.00
	*Pennisetum divisum*	0.04 ± 2.91	0–22.00
**Diet quality**	FNc	0.18 ± 0.06	0.0.79–0.034
	NDF	37.71 ± 4.63	27.80–50.70
	ADF	26.30 ± 3.02	19.8–34.3
	ADL	11.72 ± 2.20	7.8–16.30

^1^ Faecal nitrogen (FNc), neutral detergent fibre (NDF), acid detergent fibre (ADF), acid detergent lignin (ADL). “Other” refers to plant species not identified in the microhistological analysis of the faecal cuticle and “Acacias” includes *Vachellia tortilis* and *V. flava*.

## Data Availability

Not applicable.

## Supplementary Materials

The following supporting information can be downloaded at: https://www.mdpi.com/article/10.3390/ani13040567/s1, Table S1: Link of variables with the first two dimensions of the PCA.
