# Supplementary material for: Feeding Ecology of the Cuvier’s Gazelle (Gazella cuvieri, Ogilby, 1841) in the Sahara Desert
Source: Animals (Basel). 2023 Feb 6;13(4):567. doi: 10.3390/ani13040567 (PMC9951649; doi:10.3390/ani13040567)
Supplement: Supplementary file 1 [file animals-13-00567-s001.zip › animals-2058049-supplementary.pdf]

animals

MDPI

animals-2058049 Supplementary Material

Table S1. Link of variables with the first two dimensions of the PCA. For variables descriptions and variables. see text.

Variables	Dimension 1		Dimension 2	
	Correlation	*p*-value	Correlation	*p*-value
Acacias	0.30	2.24E + 04	−0.82	5.40E − 09
ADF	0.88	8.96E − 14	0.40	2.12E + 03
ADL	0.75	1.69E − 05	−0.05	7.01E + 05
Anastatica hierochuntica	−0.60	6.56E − 01	0.57	2.95E + 00
Altitud	0.31	2.09E + 04	−0.03	8.27E + 05
Annual precipitation	0.20	1.42E + 05	−0.05	6.95E + 05
Annual temperature	0.05	6.99E + 05	−0.04	7.75E + 05
Av.NDVI (per year)	0.29	2.66E + 04	−0.27	4.27E + 04
Biweekly.NDVI	0.40	2.06E + 03	−0.08	5.55E + 05
FNc	−0.55	1.06E + 01	−0.65	4.09E−02
Helianthemum lippii	−0.31	1.76E + 04	0.20	1.40E + 05
NDF	0.82	1.03E − 08	0.48	1.55E + 02
Nitraria retusa	0.13	3.25E + 05	0.29	2.66E + 04
Others	0.76	9.29E − 06	−0.36	6.05E + 03
Pennisetum divisum	0.18	1.75E + 05		
Season.NDVI	0.29	3.14E + 04	−0.16	2.22E + 05
Shannon.Ind	−0.30	2.24E + 04	0.58	2.77E + 00
